# A 3D-Printed PLA-Based Reactor with Water Cooling for Scalable and Reproducible Photocatalytic Reactions with Temperature Control

**DOI:** 10.3390/molecules31203762

**Published:** 2026-10-20

**Authors:** Denis A. Kolykhalov, Dmitry S. Gurov, Bogdan Ya. Karlinskii

**Affiliations:** 1 BioChemTech Research Center, Tula State University, Lenin pr. 92, Tula 300012, Russia; laften_71rus@mail.ru (D.A.K.); dmitriygurov2001@mail.ru (D.S.G.); 2 Zelinsky Institute of Organic Chemistry, Russian Academy of Sciences, Leninsky pr. 47, Moscow 119991, Russia

**Keywords:** 3D printing, LED, photocatalysis, PLA, reproducibility

## Abstract

Although photochemical processes are currently experiencing a renaissance and attracting significant interest from researchers worldwide, challenges with the reproducibility of reaction conditions due to various factors hinder the widespread adoption and implementation of these technologies. One such challenge is the thermal radiation emitted by LEDs, which, depending on the equipment used and its efficiency, can lead to increased or decreased heating of reaction systems and distortion of the results obtained. In this paper, we propose the use of an accessible 3D printing technique to create a hollow PLA-based photochemical reactor with the ability to incorporate flow-through cooling using water from a central water supply system. Water can be replaced with any coolant, and a chiller can be used to maintain a predetermined temperature. This reactor can be customized for any application in minutes or hours and can be scaled up or down as needed. The type of plastic used in the reactor can be easily replaced, making it a versatile and adaptable solution for any laboratory worldwide.

## 1. Introduction

Given the importance and significance of green chemistry and sustainable technologies in contemporary research, it is no surprise that photocatalysis and photoredox catalysis have emerged as some of the most rapidly evolving areas of physical and organic chemistry over the past few decades [1,2,3,4,5]. A key advantage of photoredox catalysis lies in the ability of photons to provide the energy required for target reactions to proceed, thereby eliminating the need for heating or other harsh conditions, which may be important for particularly unstable molecules [6]. The ability to conduct a variety of reactions without the need for active reducing or oxidizing agents, elevated temperatures, or harsh radiation presents a multitude of opportunities for synthesizing compounds under mild conditions, minimizing environmental impact [7,8,9,10].

**Figure 1:**
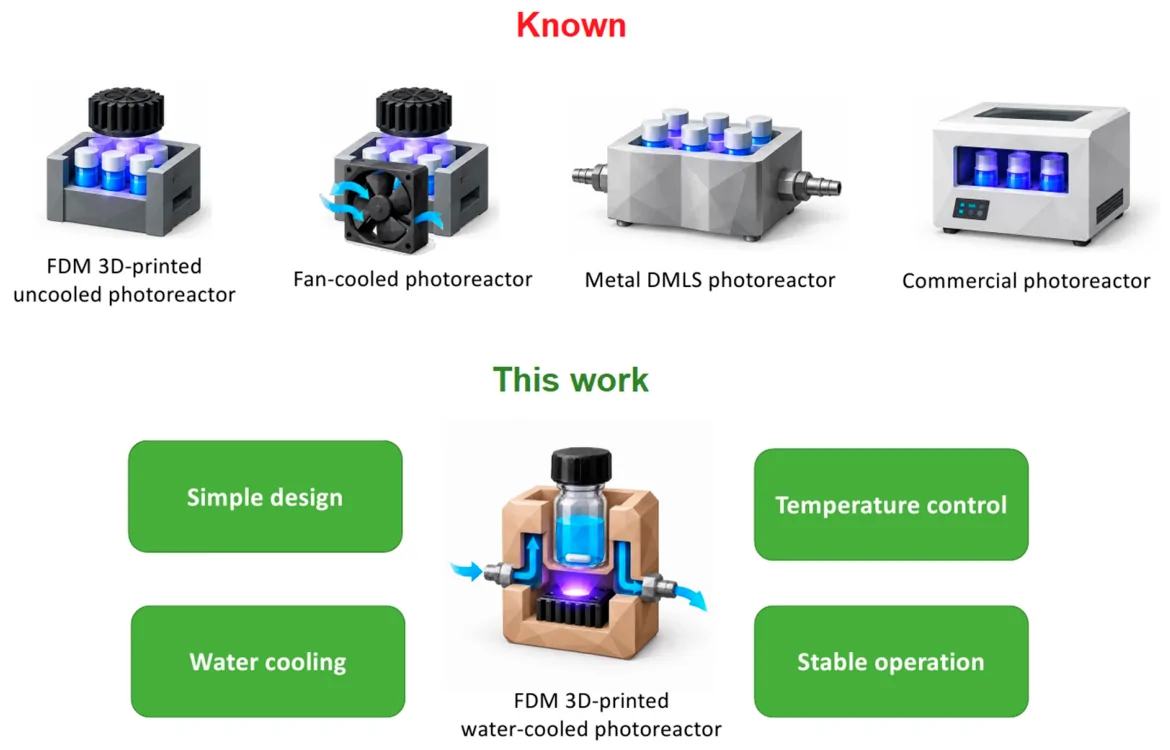
Advantages of the developed 3D-printed reactor for photochemical processes.

A key feature of photoredox catalysis, like any other new and rapidly evolving field of research, is its unconventional and nonroutine nature. Despite the great progress made in this area owing to widespread interest in research, a significant proportion of reactors and reaction systems are still assembled manually by researchers. In many laboratories, simple photoreactors without active cooling are assembled using a reaction vessel positioned at a fixed distance from one or more LEDs, often within a custom-designed housing, such as a 3D-printed enclosure or a metal casing. Unlike the traditional “flask and heater” approach, which has been successfully used in organic synthesis for hundreds of years, the unique nature of setups/reactors assembled from 3D-printed plastic [11,12,13] or metal molds [14] using various diodes and power supplies leads to a major challenge in reliably reproducing photoredox catalysis reactions [15,16,17] (Figure 1). Although the reproducibility of photochemical experiments is now widely recognized as an important issue and efforts are being made to standardize photoreactor systems, including the assessment of LED performance [16], irradiation intensity and photon flux characteristics [18,19], temperature control also requires particular attention [14]. Effective temperature regulation using air or liquid cooling should be regarded as one of the key parameters governing the stability of reaction conditions and, consequently, the reproducibility of experimental results. When high-power diodes are used without any cooling, vessel temperatures can reach 100 °C and higher, leading to drastically different results when the same reaction is performed in different settings. Since an apparent photochemical reaction may actually be catalyzed by heat emitted from the LED, the dissipation of heat from the reaction medium is particularly important.

Temperature stability during the photoreaction has also been monitored in previous studies [14,20]; however, the previously developed systems are far more difficult to replicate. The 3D printing approach is more accessible than injection molding, direct metal laser sintering (DMLS) or other high-temperature processes. This method enables the production of individual functional units without the need to machine or mill a mold.

In this Communication, we present a simple example of a 3D-printed reactor that can help mitigate the temperature issue by passing running water through the mold (Figure 1). Here, 3D printing is employed not only to create a decorative housing or a support structure for cooling elements but also to produce a single, integrated, multifunctional body that simultaneously serves as the cooling system and the housing for the reaction vessel. Given the interest and significance of research into renewable and biodegradable polymeric materials in different areas of application [21,22,23,24], polylactic acid (PLA) has been chosen as the material for the reactor. If needed, the mold can be scaled up for larger-scale reactions with any number of diodes and reaction vessels. Water can be replaced with other coolants, and a chiller system can be used in place of a water supply to conduct photochemical reactions at elevated or low temperatures.

## 2. Results and Discussion

### 2.1. Testing of the Reactor

**Figure 2:**
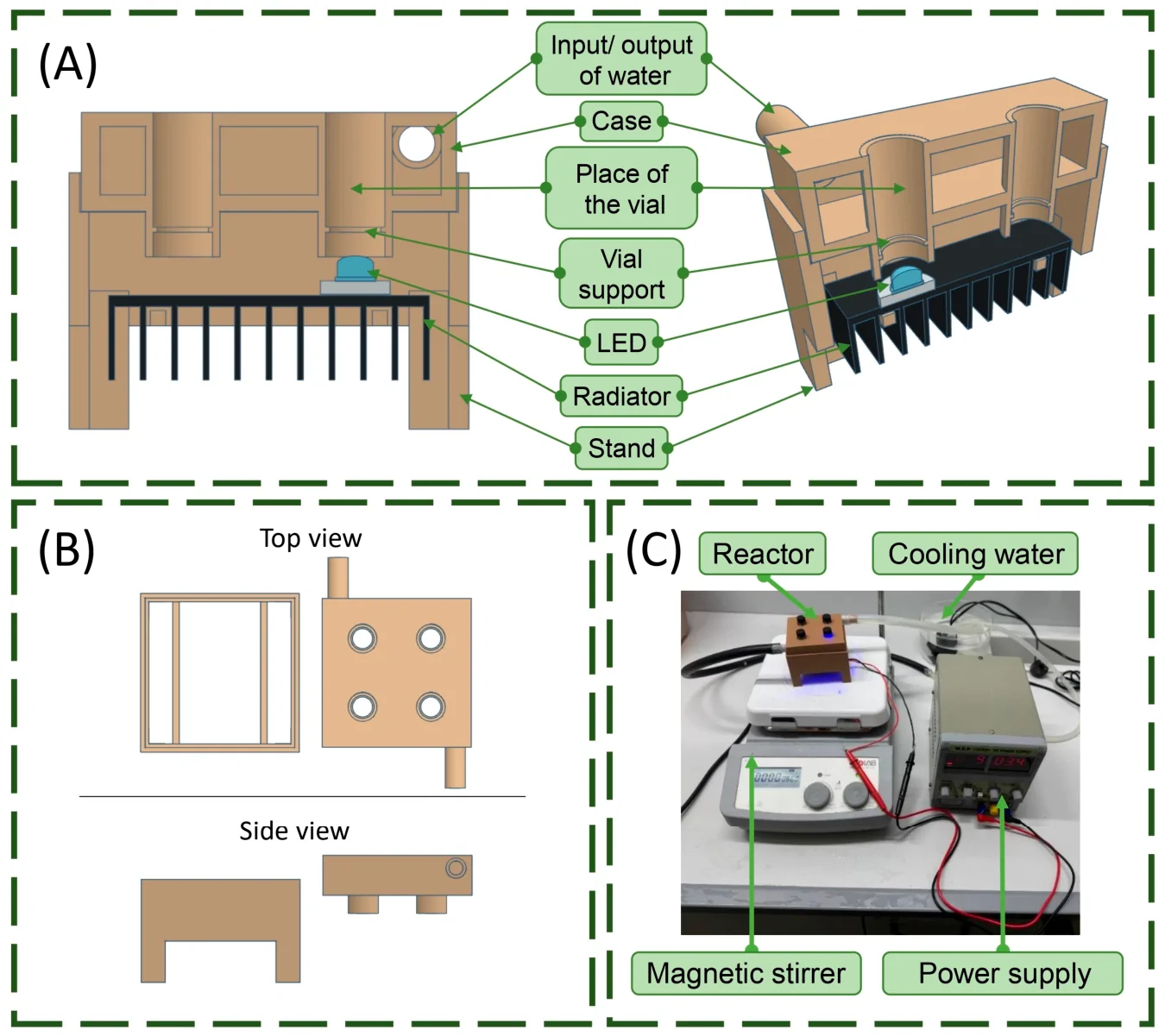
(**A**) Schematic cross-section of the reactor; (**B**) top and side views of the printed parts; (**C**) photograph of the assembled setup with the reactor, magnetic stirrer, power supply, and circulating coolant.

The development of additive manufacturing has expanded the possibilities for creating specialized laboratory equipment tailored to the requirements of specific photochemical processes. Compared with traditional manufacturing methods, 3D printing offers high production speed, low cost, and ease of scaling. On this basis, a photochemical reactor with a water-cooling system based on renewable PLA plastic was designed and manufactured for photochemical transformations under controlled conditions (Figure 2A).

In selecting the printing conditions for the photochemical reactor case, the parameters were varied with layer heights of 0.2 and 0.08 mm. While increasing the layer height clearly reduced the print time, a 0.2 mm layer height enclosure leaked slightly at the water inlet/outlet points. These defects, known as “gaps,” are associated with insufficient material adhesion [25]. For this reason, the layer height was reduced to 0.08 mm, eliminating this problem. Despite the wall of the cylindrical hole being composed of only three layers, no leaks are observed at this point, and its thin thickness allows for efficient heat dissipation from the vial.

The room temperature (RT) at the time of measurement was 23.3 °C, whereas during the photochemical reaction and LED irradiation, the temperature of the reaction medium increased to 47.6 °C without the use of a heat-removal system (Table 1). Thus, the influence of the LED leads to significant heating of the reaction mixture. This temperature increase indicates that in the absence of a heat-removal system, both photochemical and thermally induced reactions can actually occur simultaneously, which can significantly distort the interpretation of the results of photocatalytic experiments, sometimes leading to the occurrence of undesired side reactions.

The simplest cooling method involved using running water from the central water supply with a temperature of 16.1 °C. This temperature clearly varies depending on the season and how cold the water in the pipes is. In this case, the reaction medium temperature without the LED turned on was 18.8 °C, and with irradiation, it increased only to 21.1 °C. Consequently, the additional heating caused by the LED operation did not exceed 2.3 °C, which is significantly lower than that in a system without cooling.

To evaluate the ultimate cooling efficiency, precooled water at 9 °C, produced using a refrigerant, was used in a closed loop and pumped by a submersible pump. Under these conditions, the temperature in the reaction vial was 13.5 °C, and after the LED was turned on, it increased to 16.0 °C. Despite the decrease in the reaction medium temperature, the additional heating from the LED remained low (2.5 °C), demonstrating the high efficiency of the developed heat removal system.

For comparison, the possibility of using RT water circulating in a closed loop without additional continuous cooling was investigated. In this case, the reaction medium temperature stabilized at 24.7 °C. This demonstrates that even with a coolant at RT without a chiller or refrigerants, intensive liquid circulation effectively removes thermal energy from the photoreactor.

The obtained results demonstrate that the developed reactor design ensures high heat removal efficiency regardless of the coolant temperature used. Maintaining the stability of the reaction medium during LED irradiation is more important than achieving the minimum temperature. In all the liquid-cooled reactors, additional heating under the LEDs did not exceed 2.3–2.5 °C, whereas without cooling, it reached 24.3 °C. This finding indicates that virtually all the heat generated by the LEDs is effectively removed by the cooling system. Notably, using tap water from the central water supply is the most rational solution for most laboratories. This approach eliminates the need for refrigeration units or chillers and ensures a temperature regime close to RT, which corresponds to the conditions used in most photochemical syntheses. The use of cooled water is only advisable in cases where the reaction requires lower temperatures or where the temperature dependence of photochemical processes must be studied.

In the reactor presented here, the water flow rate from the submersible pump was 12.3 cm^3^/s. Consequently, the flow velocity through the 7 mm diameter outlet channel was 32 cm/s. Thus, the dynamic water pressure at the reactor outlet is 51.49 Pa. Under the conditions described, the reactor can readily withstand this internal pressure without exhibiting any visible defects or leaks. The temperature measurements indicate that even the low water flow rate effectively removes heat from the reaction vessel, thereby minimizing water consumption—particularly when main water is used. Notably, the photoreactor remains operational without defects, delamination or burnout under the influence of LEDs for 5 months, with the exception of discoloration of the plastic near the LED area. During this period, the photoreactor was used in multiple 72-h continuous runs, while water remained inside the housing during periods of inactivity.

**Figure 3:**
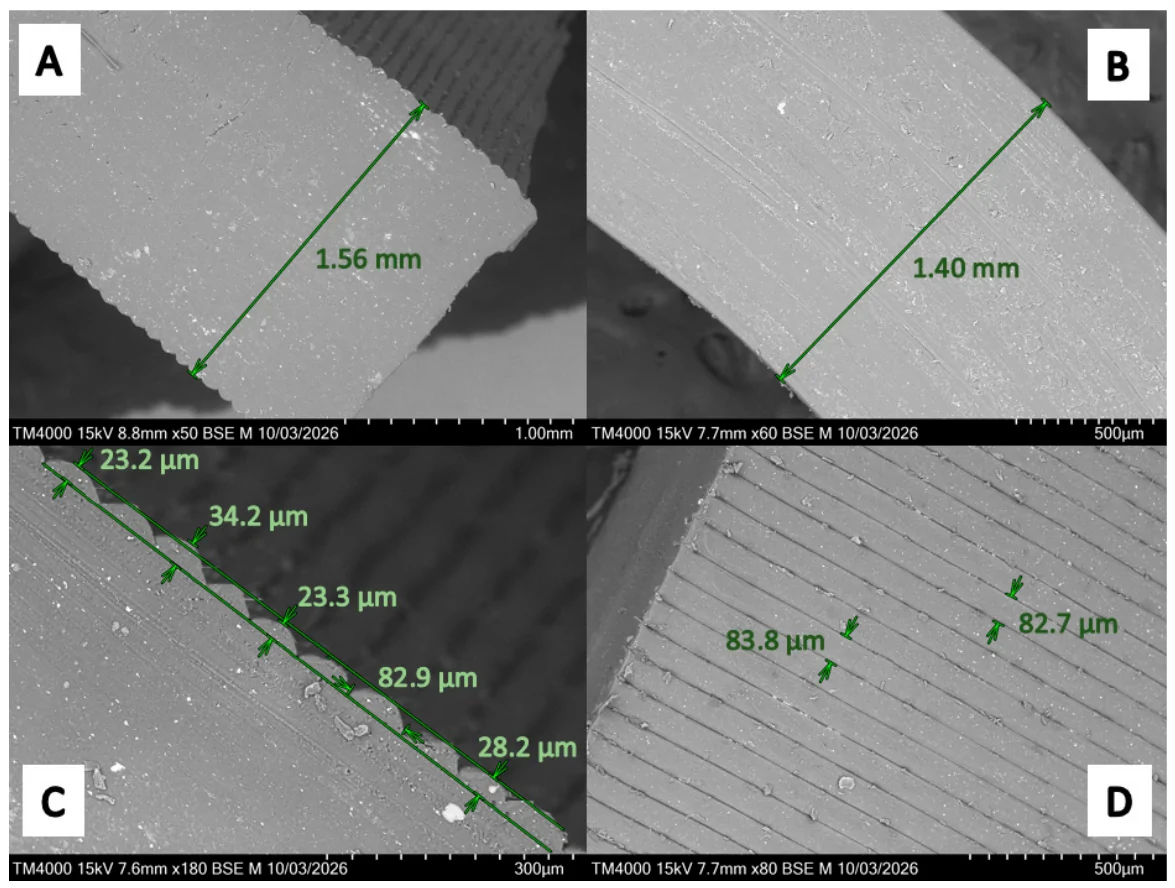
Microscopic study of the reactor wall in contact with the vial: (**A**) inner surface of the wall in a side cross-section; (**B**) inner surface of the wall in an interlaminar section; (**C**) inner surface of the wall near the contact zone with the vial (cross-sectional view); (**D**) inner surface of the wall near the contact zone with the vial (view of the layers).

Additional microscopic analysis enabled examination of the morphology and internal structure of the reactor wall near the interface with the reaction vial. The inner wall is quite uniform and lacks large voids or cracks. The wall, which consists of three layers, is approximately 1.5 mm thick (Figure 3A,B); this ensures a reliable seal for the reactor and allows efficient heat dissipation from the heating reaction vial. The height variations at the interface between the wall and the reaction vial (Figure 3C) averaged 27 µm, representing 1.74% of the total inner wall thickness. The measured layer thickness (Figure 3C,D)—based on a print setting of 80 µm—is 83 µm, which is generally consistent with expectations.

**Figure 4:**
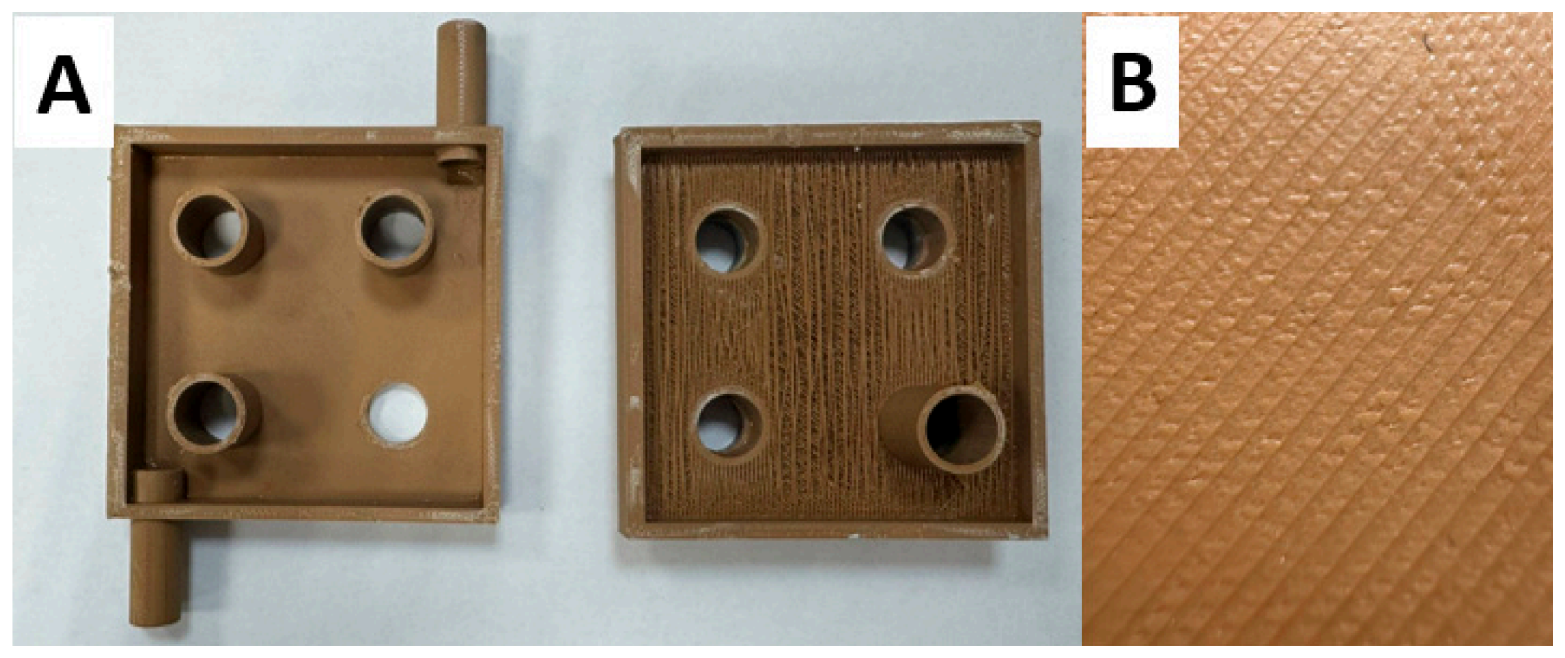
(**A**) Appearance of the reactor after continuous exposure to water for 5 months. (**B**) Close-up view of the appearance and texture.

Additionally, the internal condition of the reactor was examined after five months of exposure to water. To this end, the reactor was split lengthwise into two parts (Figure 4). The internal state of the reactor after five months of exposure to water is shown in Figure 4A. The deformed wall visible on the right in Figure 4A resulted from printing the reactor interior without support; however, layering effectively compensates for this deformation, allowing the reactor to operate without leaks. Furthermore, the morphology of the reactor (Figure 4B) remained virtually unchanged after prolonged contact, with extrusion marks still clearly visible. It can be said that the high level of quality and precision of modern 3D printers enables the design and production of high-precision, hermetically sealed tools for practical applications in chemistry.

### 2.2. The Validation of the Developed Reactor for Use in Photochemical Processes

After developing the reactor and evaluating the effectiveness of the cooling system, experimental testing of the developed reactor was conducted under organic synthesis conditions. For this purpose, four model photochemical reactions with different classes of sensitizers were performed, including those using LEDs with different wavelengths. The selected reactions are intended to demonstrate the applicability of the reactor under various photochemical conditions—the use of diodes of different wavelengths and various catalysts—in both homogeneous and heterogeneous systems and with varying irradiation times.

**Scheme 1:**
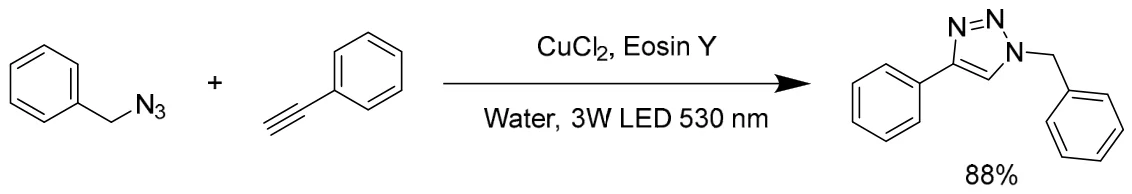
Photoinduced copper-catalyzed azide–alkyne cycloaddition reaction.

A copper-catalyzed azide–alkyne cycloaddition (CuAAC) reaction between benzyl azide and phenylacetylene to form the corresponding 1,4-disubstituted 1,2,3-triazole was chosen as one of the model reactions (Scheme 1). This reaction is an example of a widely used “click” chemistry process and typically occurs in the presence of catalytically active copper(I) compounds [26,27].

CuCl_2_ was used as an accessible copper source. The active form of the Cu(I) catalyst was generated directly in the reaction medium by the photochemical reduction of Cu(II). Eosin Y was used as a photosensitizer. When illuminated with green light, Eosin Y entered an electronically excited state and participated in single-electron transfer, reducing Cu(II) to Cu(I). The resulting Cu(I) species then initiated the cycloaddition of the terminal alkyne to the organic azide.

The choice of this reaction for testing the developed reactor was driven by the need to simultaneously control two factors—irradiation intensity and reaction medium temperature. Cu(I) generation directly depends on the efficient excitation of Eosin Y by green light, whereas uncontrolled heating can alter the rate of both the photochemical stage and the subsequent catalytic conversion. The use of the developed liquid-cooled reactor allowed the reaction to be conducted at a stable temperature, thereby reducing the contribution of thermal factors. This suggests that the reaction proceeds primarily through the photochemical formation of the catalytically active form of Cu(I) rather than through heating of the reaction mixture.

**Scheme 2:**
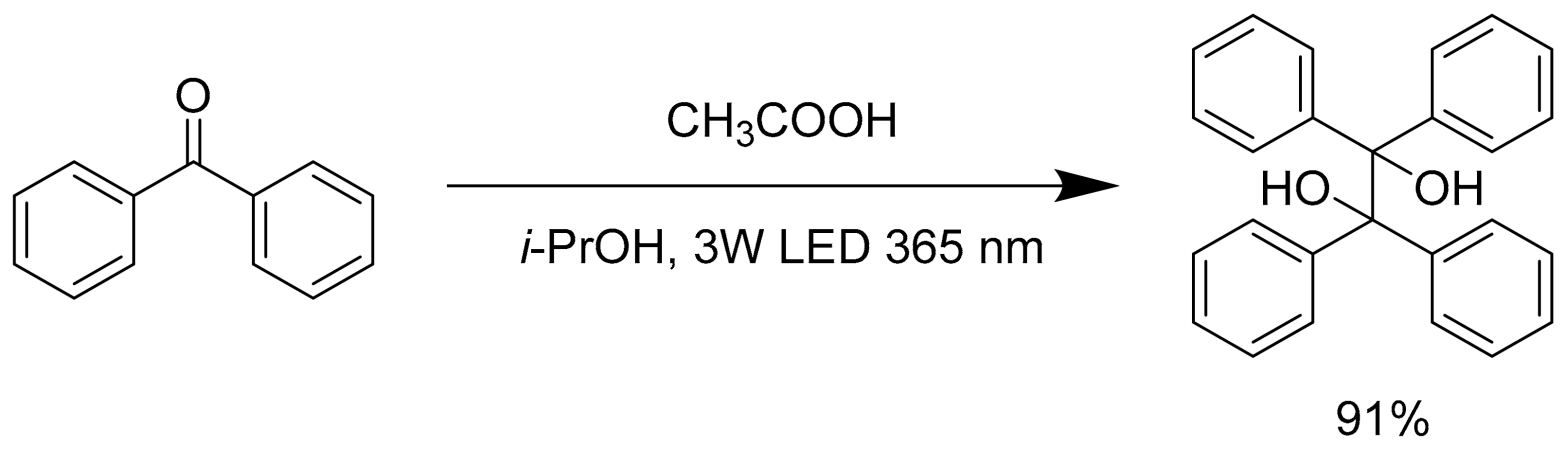
Photochemical pinacolization of benzophenone.

The photochemical pinacolization of benzophenone to form benzopinacol [28] was chosen as the second model reaction (Scheme 2). This reaction is a classic example of the photochemical reduction of a carbonyl compound and is traditionally carried out by irradiating a solution of benzophenone in isopropyl alcohol. In this process, isopropanol simultaneously functions as a solvent and a hydrogen atom donor and is oxidized to acetone.

After the first irradiation cycle was complete, the liquid phase was carefully transferred to another vial, separated from the precipitated product, and irradiated again for 16 h. The procedure of sequentially separating the crystals and reirradiating the mother liquor was continued until benzopinacol precipitation ceased. This approach allowed for more complete conversion of the benzophenone remaining in the solution and simultaneously shifted the process toward product formation because of its gradual removal from the reaction medium. Sequential irradiation of the mother liquor facilitates more complete utilization of the starting benzophenone and increases the overall yield of the product.

This reaction is indicative of testing the developed reactor, as it requires prolonged exposure to ultraviolet light. Without cooling, prolonged operation of the LED could lead to significant heating of the solution, changes in the solubility of benzophenone and benzopinacol, and an increase in the rate of thermal byproduct formation. The use of a liquid cooling system ensured stable irradiation conditions throughout each 16-h cycle and facilitated reproducible precipitation of the crystalline product.

**Scheme 3:**
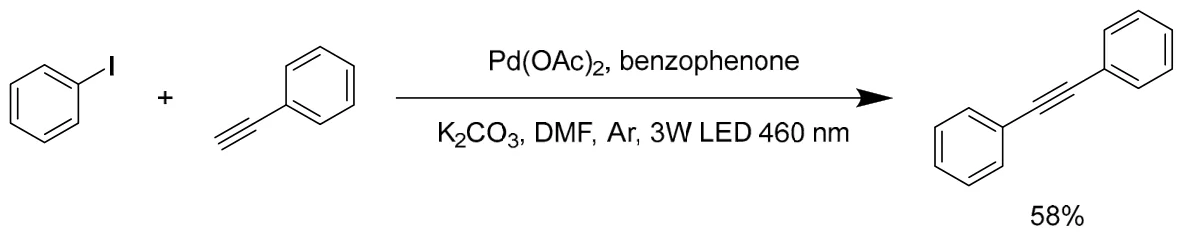
Photoinduced Pd-catalyzed Cu-free Sonogashira reaction.

The third model reaction involved the Pd-catalyzed cross-coupling of aryl halides with terminal alkynes (the Sonogashira reaction), which occurs under the influence of visible light without the use of traditional copper cocatalysts (Scheme 3). This reaction is among the most popular methods for forming C–C bonds in the synthesis of pharmaceutical compounds, natural products, and functional organic materials [29].

In contrast to the classical Sonogashira reaction, in this method, the formation of the catalytically active palladium complex is initiated photochemically [30]. Under the influence of blue light, benzophenone is transformed into an excited state and participates in electron transfer, resulting in the reduction of palladium and the regeneration of the active catalytic form Pd(0). The standard catalytic cycle then proceeds, including the oxidative addition of the aryl halide to palladium species, coordination and deprotonation of the terminal alkyne in the presence of potassium carbonate, subsequent transmetallation and reductive elimination with the formation of a new C–C bond. The use of the photocatalytic mode eliminates the need for a copper cocatalyst, which reduces the likelihood of a side reaction, such as the homocoupling of alkynes (Glaser reaction) [31].

**Scheme 4:**
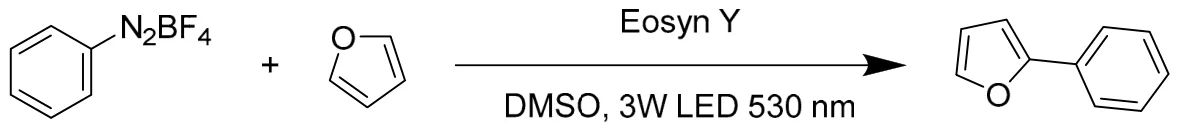
Photoinduced arylation of furan with Eosin Y as a photosensitizer.

As a fourth model reaction, a photocatalytic furan arylation reaction was investigated using aryldiazonium salts as a source of aryl radicals [32]. Reactions of this type are widely used for the selective functionalization of electron-rich heterocycles and are convenient models for assessing the efficiency of photochemical reactors (Scheme 4).

The reaction mechanism is based on the photocatalytic cycle of Eosin Y. When the photocatalyst is irradiated with green light, it becomes excited and undergoes one-electron reduction of the aryldiazonium salt. This results in the formation of an aryl radical with the simultaneous release of molecular nitrogen. The aryl radical then attaches to a furan molecule to form a new carbon radical, which, after oxidation and deprotonation, is converted into the corresponding arylated product. Thus, light is necessary for the continuous generation of reactive radical intermediates.

To evaluate the effects of temperature and irradiation, the reaction was run in four different modes, including active cooling of the circulating water with a refrigerant. The highest product yield (65%) was achieved with green light irradiation while the reaction medium temperature was maintained at ≈15 °C (Table 2). When the reaction was run under irradiation at ≈25 °C, the yield decreased slightly to 61%, indicating a small but noticeable dependence of the process efficiency on temperature. In the absence of irradiation, the reaction essentially stopped at ≈15 °C, and the product yield did not exceed 5%. When the reaction was run without light at ≈25 °C, the yield increased to 22%, which is likely due to the slow thermal decomposition of the aryldiazonium salt with the formation of aryl radicals.

The obtained results clearly demonstrate that photochemical activation is the determining factor in this reaction. Increasing the temperature in the absence of light only partially compensates for the lack of radical photogeneration and does not allow the yields observed with irradiation to be achieved. Moreover, decreasing the temperature while maintaining a constant irradiation intensity leads to an increase in the product yield, which may be due to a decrease in the rate of side processes, such as the thermal decomposition of aryldiazonium salts, radical recombination, or the formation of byproducts. This model reaction particularly well demonstrates the advantages of the developed photochemical reactor. Owing to the use of an efficient liquid cooling system, it was possible to conduct the reaction at a low, controlled temperature, eliminating heating of the reaction mixture with an LED light source. This made it possible to separate the effects of temperature and irradiation intensity on process efficiency and confirmed that maintaining a low and stable temperature contributes to increased selectivity of photocatalytic arylation.

Thus, the results of the model reactions confirmed that the developed reactor provides not only the required irradiation intensity but also effective temperature control of the reaction medium, which is a key factor in the reproducibility of photochemical processes. The absence of significant overheating during operation allows the proposed design to be considered a universal platform for conducting a wide range of photochemical reactions in laboratory practice.

### 2.3. Possible Variations of the Presented Reactor

**Figure 5:**
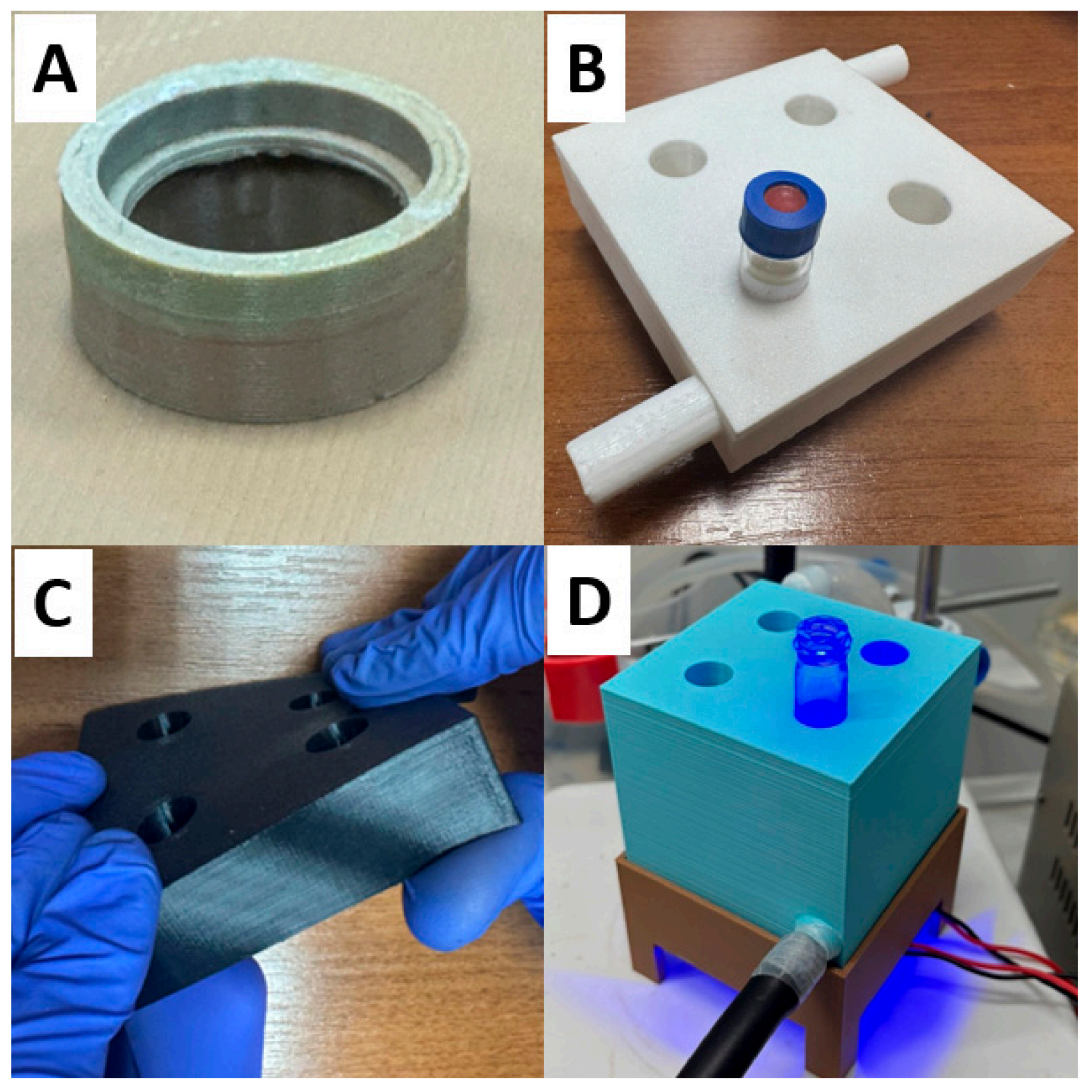
(**A**) Color change of the reactor near the LED; (**B**) white PETG reactor; (**C**) flexible black TPU reactor; (**D**) plug-and-play reactor replacement for Duran GL14 tubes, maintaining LED positioning.

PLA is among the most accessible and widely used types of plastic in 3D printing. Furthermore, it is biodegradable and renewable. The material remains functional, although the color of the plastic near the LED may change because of the discoloration of the organic dye (Figure 5A). However, it is also possible to use other materials for printing the reactor. For instance, polyethylene terephthalate glycol plastic (PETG) (ABS Maker, Zhangjiagang, China) (Figure 5B) was tested for printing the reactor case and the nonstandard thermoplastic polyurethane (TPU-95A) (Kingroon, Shenzhen, China) (Figure 5C), which can only be printed using a “direct extrusion” system. The use of TPU plastic solves the problem of reactor brittleness. It was decided not to print the case using acrylonitrile butadiene styrene (ABS) plastic, as printing with ABS is a nonstandard task requiring a printer equipped with a temperature-controlled chamber to prevent layer delamination. Additionally, the room housing the printer must be well ventilated, as the ABS printing process releases toxic fumes [33]. Another disadvantage of ABS plastic is its degradation and deterioration upon exposure to UV irradiation [34], which acts as a limiting factor for photochemical reactions driven by UV light.

The reactor was also modified to accommodate standard Duran GL14 tubes (13 mm diameter, Mainz, Germany). A “plug-and-play” reactor replacement system was implemented (Figure 5D), requiring only the connection of water inlet and outlet lines and the placement of the reactor on the stand, as the LED arrangement remained unchanged. The reactors were tested using the “LED on, RT water” setup. The temperature was measured according to the procedure described in Section 3.2. The temperature of the reactor printed from PLA for Duran GL14 tubes was 25.2 °C; for 1.5 mL PETG vials, it was 25.3 °C; and for TPU vials, it was 27.2 °C.

The results demonstrate that the reactor we have presented can serve as a platform for further modification to suit specific photochemical applications—enabled by varying the types of filaments and reaction vessels employed—while its excellent seal integrity allows the printed housing to function directly as a flow-through vessel.

### 2.4. Comparison with Existing Photoreactor Systems

The developed photoreactor combines a simple design, ease of fabrication via fused deposition modeling (FDM) printing, and effective temperature control of the reaction medium. A key feature is the integration of the vial holder and the water-cooling jacket into a single component, which reduces the number of individual parts and simplifies assembly and operation. Tap water can be used for cooling, and the temperature rise of the reaction mixture during irradiation is only ~2.3–2.5 °C. Furthermore, the reactor was operated without leaks for five months, confirming the practical viability of the chosen design (Table 3).

Compared with more complex systems fabricated from metal using additive manufacturing, the proposed approach is significantly simpler and more accessible for a standard laboratory. Metal photoreactors provide efficient heat exchange and can be adapted for heating, parallel experiments, and scale-up; however, their fabrication is technically more complex and less accessible. Other FDM-printed PLA photoreactors [13] also offer ease of fabrication and adaptability to vessels of various sizes, yet active cooling may be directed primarily at the LED, without directly removing heat from the reaction medium.

Portable FDM-printed systems have also been designed, for instance, for photochemical water purification as well as more versatile units capable of operating in both batch and continuous flow modes, enabling parallel screening and simplifying scale-up [18]. Despite the advantage of controlling the reaction temperature, the developed reactor has a limited functional scope compared to these solutions: it operates only in batch mode and each reaction vessel requires a separate LED. Moreover, the bottom-mounted light source allows the reaction to take place only in close proximity to the LED.

Thus, the proposed photoreactor is a simple and easily modifiable system for laboratory-scale photochemical synthesis, combining the accessibility of FDM printing with direct cooling of the reaction medium. Compared with commercial units, it offers a lower degree of automation and standardization; however, it does not require expensive proprietary equipment and allows for greater flexibility in design modification.

## 3. Materials and Methods

### 3.1. 3D Printing of the Plastic Reactor

The reactor was printed using a “QIDI Q1 Pro” FDM 3D printer (Shenzhen, China) and a PLA+ filament from Shenzhen Esun Industrial Co., Ltd. (eSUN, Shenzhen, China). In accordance with the manufacturer’s specifications, the deformation temperature of this material was 54 °C, whereas the printing temperature was 210 °C. Additional material characteristics are presented in Table 4. PLA was selected because of its availability, low cost, good printability, dimensional accuracy, and suitability for rapid prototyping using widely available FDM equipment.

The developed photoreactor is a hollow rectangular parallelepiped (74 × 74 × 20 mm) with cylindrical holes (11.4 mm) through it, which accommodate the reaction vessel (Figure 2A,B). The diameter of the water inlet/outlet tube is 7 mm. This example uses a 1.5 mL vial, but the model can be adapted to accommodate a vessel of any size. A small protrusion inside the cylindrical hole prevents the bottle from falling out. By adjusting the position of this protrusion, the distance from the LED to the bottom of the vial can be regulated. Coolant access to the interior of the housing is provided through an inlet and outlet.

The model was designed in the Tinkercad (https://www.tinkercad.com). The standard “PLA printing conditions” with a 0.08 mm layer thickness are presented in Table 4. “Standard PLA printing conditions” refers to the settings provided by the printer manufacturer and the QIDI Studio 2.7.2.10 software for the PLA material and a layer height of 0.08 mm. The preset for the specific PLA filament and the reactor print settings are provided in the Supplementary Materials as files ready for import into the software. However, it should be noted that the printing conditions vary for each 3D printer; therefore, it is best to rely on the manufacturer’s recommendations for each specific model. For this reason, printing conditions for PETG and TPU-95A are not provided here. Support-free printing was used for the construction of the interior part of the reactor, and the input/output port and vial support were printed with the support. The reactor structure can be found in the attached .stl files.

The case is installed on a special stand with fins for mounting an aluminum heatsink. The distance between the LED and the bottom of the 1.5 mL vial is 3 mm. 3 W LEDs (KolGur, Bogoroditsk, Russia) are installed in the heatsink using thermal grease. This ensures passive cooling of the LEDs and extends their lifespan. A laboratory power supply (output voltage 0–15 V, output current 0–2 A; W.E.P., Shenzhen, China) is used as the power source. The system can be cooled in several ways: using running water from the central water supply system or using a water pump that continuously circulates water in a closed loop. An external chiller with any available refrigerant can also be used to maintain the reaction medium temperature. This reactor can be installed on a magnetic stirrer to ensure mixing of the reaction medium (Figure 2C).

### 3.2. Reactor Testing

The process of heat transfer from the heating reaction medium to the photochemical reactor vessel was studied using tap water from the central water supply (a), water cooled by ice-based refrigerants, replaced every 15 min (b), and circularly pumped RT water (c). The temperatures were measured no earlier than one hour after the photochemical unit was started, as the reaction medium temperature reached a steady state during this time [14]. To maintain a minimum cooling liquid temperature, the coolant was replaced every 15 min. To measure the temperature, the thermocouple was placed in a test tube containing 1 mL of water, touching the bottom, where the hottest zone is located. The average temperature measurements are presented in Table 1.

The dynamic water pressure at the reactor outlet was calculated using Formula (1):

*P* = (*ρV*^2^)/2 (1)

where *P*—dynamic pressure at the reactor outlet, Pa; *ρ*—water density, kg/m^3^; *V*—flow velocity, m/s.

The flow velocity was calculated using Formula (2):

*V* = *Q/S* (2)

where *Q*—water consumption, cm^3^/s; *S*—cross-sectional area at the reactor outlet, cm^2^.

A microscopic study was carried out using a Hitachi TM4000 tabletop scanning electron microscope (Tokyo, Japan). Images were obtained in reflected electron BSE mode at an accelerating voltage of 15 kV and at a working distance of 7–9 mm. For microscopic examination, the reactor was cut and delaminated using a sharp scalpel.

### 3.3. Photochemical Reactions

Reaction 1, photoinduced CuAAC: The reactions [26] were carried out in a 1.5 mL glass vial. Benzyl azide (50 mg, 0.375 mmol), phenylacetylene (46 mg, 0.450 mmol), and 1 mL of water were added to the vial. Eosin Y (2.4 mg, 1 mol%) and CuCl_2_ (0.504 mg, 1 mol%) were then added; the resulting mixture was stirred in a sealed vial under irradiation with a green LED (530 nm) for 2 h. After completion of the reaction, the product was isolated by column chromatography to obtain a white crystalline substance (78 mg, 88%).

Reaction 2, pinacolization: In a 1.5 mL vial, benzophenone (150 mg, 0.820 mmol) was dissolved in isopropanol (850 μL), and a drop of acetic acid (2 μL) was added [28]. The vial was then placed in a reactor and irradiated with 365 nm light. After 16 h, benzopinacol product crystals were observed at the bottom of the vial. The solution was then transferred to another 1 mL vial and placed back in the reactor for 16 h. This procedure was repeated until the benzopinacol crystals stopped precipitating. The crystals were subsequently washed with isopropyl alcohol and dried. The final product was transparent crystals, with a total yield of 137 mg (91%).

Reaction 3, copper-free Sonogashira coupling: To a 1.5 mL vial containing Pd(OAc)_2_ (2 mol%, 2.2 mg), K_2_CO_3_ (0.735 mmol, 101 mg), benzophenone (10 mol%, 9 mg) and 1 mL of *N*,*N*-dimethylformamide (DMF), iodobenzene (0.490 mmol, 100 mg) and phenylacetylene (0.735 mmol, 81 μL) were added [30]. The reaction mixture was irradiated with a 3 W blue LED (460 nm) for 6 h. After the reaction was complete, distilled water (2 mL) was added to the reaction mixture, and the crude product was extracted with ethyl acetate and further purified by column chromatography using petroleum ether and ethyl acetate (8:2) as the eluent. The product was white crystals with a yield of 51 mg (58%).

Reaction 4, arylation with diazonium salt: Aryldiazonium tetrafluoroborate salt was preliminarily synthesized according to the procedure [32]. Eosin Y (0.05 mmol, 4 mg), aryldiazonium tetrafluoroborate (0.520 mmol, 100 mg) and furan (5 mmol, 381 μL) were dissolved in dry DMSO (1 mL) in a 1.5 mL vial. The vial was irradiated with a green LED (530 nm). After 2 h of irradiation, the reaction mixture was transferred to a separatory funnel, diluted with diethyl ether and washed with 15 mL of water. The aqueous layer was subsequently washed three times with diethyl ether. The combined organic layers were dried over MgSO_4_, filtered and concentrated in vacuo. The crude product was purified by flash column chromatography using petroleum ether/ethyl acetate as the eluent. The product was a colorless liquid with a yield of 46 mg (61%).

## 4. Conclusions

Given the significant interest of researchers in the field of photoredox catalysis and the persistent issue of reproducibility of reactions in different laboratory-constructed catalytic setups, it is crucial to unify or, in some way, standardize using equipment and approaches. Our paper presents a simple and sustainable 3D-printed photochemical reactor that minimizes the effect of temperature on the reaction, which is a significant confounding factor due to imperfections and the inefficient nature of LEDs. This hollow PLA-based reactor has the ability to accommodate flow-through cooling using water from a central water supply. Water can be substituted with any coolant, and a chiller can maintain a predetermined elevated or lowered temperature. The temperature variation of the reaction medium under the influence of LED irradiation did not exceed 2.5 °C compared with that of the control sample, which was not exposed to LED irradiation.

A microscopic examination of the reactor wall revealed that there was no evidence of any damaging effect from light or water on the inside or outside of the photoreactor. Using the example of four representative photocatalytic reactions involving different types of organic and metal complex photosensitizers, we demonstrated the feasibility of the developed approach for use in laboratory settings. The reactor can be customized for various applications in minutes or hours, and it can be scaled up or down as needed. The type of plastic used in the reactor is easily replaceable, making it a flexible and adaptable solution for synthetic photocatalytic laboratories worldwide.

## Figures and Tables

**Table 1:** Measured values of the temperature of the cooling liquid and reaction medium.

Conditions	Temperature, °C
RT	23.3
LED on, no water ^1^	47.6
Central water supply (a)	16.1
LED off, central water supply ^1^ (a)	18.8
LED on, central water supply ^1^ (a)	21.1
Cooled water (b)	9.0
LED off, cooled water ^1^ (b)	13.5
LED on, cooled water ^1^ (b)	16.0
LED on, RT water ^1^ (c)	24.7

^1^ Temperature inside a 1.5 mL vial. (a) Cooling the reactor with cold tap water; (b) cooling the reactor with circulating water using replaceable refrigerants; (c) cooling the reactor with circulating RT water without refrigerants.

**Table 2:** Efficiency of arylation with and without LED irradiation at different temperatures.

Conditions	Product Yield, %	
	~15 °C	~25 °C
LED off	<5	22
LED on	65	61

**Table 3:** Comparison of the developed reactor with other examples.

Advantages	Disadvantages	Reference
Produced FDM printing using PLA plastic Simple design The vial holder and the water-cooling jacket are a single component Capability to operate using central water supply The temperature rise during irradiation is 2–3 °C Leak-free reactor operation for five months Open platform for modification Plug-and-play reactor vessel replacement	Batch reactor One LED per reaction vessel	This work
Metal versions ensure efficient heat exchange The ability to heat the system	Metal additive manufacturing is significantly less accessible	[14]
A simple PLA photoreactor fabricated using the FDM method Quickly adjustable for vessels of various sizes Flow-through and batch reaction modes	The need for active cooling of the LED There is no heat removal from the reaction medium	[13]
Portable photoreactor for water purification FDM 3D-printing photoreactor Possibility of using UV or visible irradiation	Single-section building	[11]
Batch and continuous flow modes Simple path to scaling	High cost of Kessil LEDs (Taiwan)	[18]
Supplied as ready-to-use, calibrated systems High result reproducibility Well-defined irradiation geometry Precise control of light intensity and temperature Higher level of safety High-quality technical documentation	Significantly higher cost Difficulties in making design changes Dependence on proprietary equipment or components	Commercial photoreactors

**Table 4:** Basic plastic characteristics and parameters of reactor printing.

Parameters	Value
Manufacturer	Shenzhen Esun Industrial Co., Ltd.
Plastic	PLA+
Tensile strength, MPa	36 *
Flexural strength, MPa	56 *
Elongation at break, %	3 *
Flexural modulus, MPa	2874 *
Izod impact strength, J/m^2^	2.2 *
Melt flow index, g/10 min	3.7 (190 °C/2.16 kg) *
Density, g/cm^3^	1.24 *
Deflection temperature, °C	54 *
Filament diameter, mm	1.75 *
Print bed temperature, °C	60
Nozzle temperature, °C	210
Nozzle diameter, mm	0.4
Layer height, mm	0.08
Extrusion width, mm	0.42
Filling density, %	100
First layer printing speed, mm/s	50
Print speed of outer perimeter, mm/s	200
Print speed of the inner perimeter, mm/s	350
Case printing time, h	3.3
Weight of the case, g	52.9
Stand printing time, h	4.1
Weight of the stand, g	39.5

* Information taken from the filament manufacturer.

## Data Availability

The original contributions presented in this study are included in the article/Supplementary Materials. Further inquiries can be directed to the corresponding author.

## Supplementary Materials

The following supporting information can be downloaded at: https://www.mdpi.com/article/10.3390/molecules31203762/s1, parameters for 3D printing: “PLA profile.json”, “PETG profile.json”, “TPU 95A profile”, and “Print profile.json”; models of photoreactors: “Case for 1.5 mL vials.stl”, “Case for 6 mL tubes.stl”, and “Stand.stl”.

## Abbreviations

The following abbreviations are used in this manuscript:

CuAAC	Copper(I)-catalyzed Azide–Alkyne Cycloaddition
FDM	Fused Deposition Modeling
LED	Light-emitting Diode
PLA	Polylactic Acid
RT	Room Temperature
